# Real-World Implementation and Evaluation of a Digital Health Platform for Children Living With Type 1 Diabetes: Protocol for a Real-World Implementation Study

**DOI:** 10.2196/107634

**Published:** 2026-10-30

**Authors:** Shruti Muralidharan, Molly Sweeney Magee, Kimberly Charbonneau, Simona McKee, Carlie Zachariuk, Sukhpreet K Tamana, Gonzalo Dominguez-Menendez, Tricia S Tang, Elizabeth M Borycki, Andre W Kushniruk, Wyeth W Wasserman, Jeffrey N Bone, Matthias Görges, Shazhan Amed

**Affiliations:** 1 Department of Pediatrics, University of British Columbia, Vancouver, BC, Canada; 2 BC Children's Hospital Research Institute, A4-196 938 West 28th Avenue, Vancouver, BC, V5Z 4H4, Canada; 3 Haibu Health, Vancouver, BC, Canada; 4 Department of Clinical Health Psychology, Max Rady College of Medicine, University of Manitoba, Winnipeg, MB, Canada; 5 School of Health Information Science, University of Victoria, Victoria, BC, Canada; 6 Department of Medical Genetics, University of British Columbia, Vancouver, BC, Canada; 7 Centre for Molecular Medicine and Therapeutics, University of British Columbia, Vancouver, BC, Canada; 8 Department of Statistics, University of British Columbia, Vancouver, BC, Canada; 9 Department of Anesthesiology, Pharmacology and Therapeutics, University of British Columbia, Vancouver, BC, Canada

**Keywords:** type 1 diabetes, pediatric diabetes, digital health, implementation science, effectiveness-implementation hybrid trial, continuous glucose monitoring, patient-centered care, clinical workflow, real-world evaluation

## Abstract

**Background:**

Digital platforms that integrate continuous glucose monitoring, patient-generated data, and clinical decision support could enable more proactive pediatric type 1 diabetes (T1D) care. Sustained uptake of digital platforms in routine practice is limited, and the evidence of effectiveness is mixed. Implementation science approaches are needed to clarify how context, workflows, and strategies shape adoption and outcomes. Haibu Diabetes is a new platform that brings together wearable, patient-reported, and clinical diabetes data to generate personalized, data-driven insights for patients and clinicians.

**Objective:**

This study evaluates the clinical effectiveness and implementation processes of Haibu Diabetes in routine pediatric T1D care using a hybrid design guided by an Implementation Research Logic Model.

**Methods:**

We will conduct a nonrandomized, quasi-experimental, type 2 hybrid implementation-effectiveness trial at a hospital that provides multidisciplinary pediatric diabetes care. Youth aged 3 to less than 18 years with T1D and caregivers will be onboarded to Haibu Diabetes as part of routine workflows; health care providers will use a clinician dashboard to review data, assign tasks/forms, and communicate asynchronously. Effectiveness analyses will compare Haibu Diabetes users to a matched control group drawn from the BC Pediatric Diabetes Registry. Effectiveness outcomes are hemoglobin A_1c_, continuous glucose monitoring device-derived time in range over 12 months, and diabetes self-care behaviors. Implementation outcomes will be assessed using mixed methods guided by an Implementation Research Logic Model that links determinants, implementation strategies, mechanisms, and outcomes. These outcomes will include reach, adoption, feasibility, acceptability, and maintenance. Health care improvement outcomes will include patient and caregiver experience, clinician experience, and operational workflow indicators.

**Results:**

Study funding commenced in January 2023. Patient recruitment began in September 2026, and 7 participants had consented as of October 2026. Data collection is anticipated to conclude by December 2027, with analysis to follow. Study findings are expected to be published in summer 2028.

**Conclusions:**

This trial will generate real-world evidence on both effectiveness and the conditions required to implement and sustain a data-integrating digital platform in pediatric T1D care, informing scale-up and optimization.

## Introduction

### Problem Description

Digital technology for diabetes management is becoming increasingly prevalent and currently offers opportunities to integrate continuous glucose monitoring (CGM) and other patient-generated health data into routine care for children and adolescents with type 1 diabetes (T1D). When embedded in clinical workflows, these tools can support clinical decision-making and enable data aggregation and visualization, self-management support, and patient-clinician communication [1-4]. Population-management dashboards and data-informed workflows may enable more proactive care, though reported benefits vary by setting and implementation approach [5-8].

### Available Knowledge

Despite ongoing innovation in digital technology for diabetes management, uptake and sustained integration in routine clinical practice remain limited. This reflects persistent implementation and contextual challenges that constrain its effective use in real-world settings [2]. These challenges operate at multiple levels: clinicians may encounter documentation burden, workflow disruption, and poor usability of digital systems [2,9], while patients and caregivers may face inequitable access to diabetes technologies, limited access to smartphones or reliable internet, low digital literacy, and concern that digital tools may weaken relational connection in care [10-13]. Moreover, evidence of consistent improvement in clinical and patient-reported outcomes (eg, hemoglobin A_1c_ [HbA_1c_], time in range [TIR], and diabetes distress) attributable to the use of digital diabetes management tools remains inconsistent across interventions and real-world contexts [14,15]. Collectively, these findings point to a need for implementation science–informed approaches that can systematically identify determinants, support selection of implementation strategies, and guide evaluation of both clinical and implementation outcomes.

### Rationale

The Implementation Research Logic Model (IRLM) [16] is one such approach. It provides a structured, theory-informed framework that links implementation determinants, strategies, mechanisms of action, and outcomes, enabling systematic planning and evaluation of implementation processes [16]. In this study, we use the IRLM to make explicit the contextual factors and implementation processes expected to influence adoption, integration, and sustainment of Haibu Diabetes—a new digital platform that integrates wearable, patient-reported, and clinical diabetes data to generate personalized, data-driven insights for patients and clinicians (Haibu Health Ltd).

Prior to the current study, we piloted an early prototype of Haibu Diabetes (TrustSphere) to support collaborative care for youth with T1D [17]. The pilot demonstrated feasibility and potential benefit, while also identifying opportunities for refinement that informed the next generation of Haibu Diabetes [17]. To evaluate this new version and address the broader knowledge gap regarding how and under what conditions digital tools can be effectively implemented into routine care, we designed the Intelligent Care Platform trial, a type 2 hybrid implementation-effectiveness trial [18] at a hospital that delivers multidisciplinary pediatric diabetes care in British Columbia, Canada. Guided by the IRLM, our objective is to evaluate both the clinical effectiveness of Haibu Diabetes and the processes through which it is implemented and integrated into diabetes clinic workflows, including the determinants, strategies, and mechanisms expected to shape implementation outcomes within this setting. This paper describes the trial design and evaluation plan, guided by the IRLM. This protocol is reported in accordance with the SQUIRE 2.0 (Standards for Quality Improvement Reporting Excellence) guidelines (Checklist 1) [19].

### Specific Aims

This study has 3 research aims.

- Aim 1: To evaluate the effectiveness of Haibu Diabetes in improving clinical outcomes in T1D management by comparing HbA_1c_, TIR, and diabetes self-care between Haibu Diabetes users and nonusers.
- Aim 2: To assess the reach, feasibility, adoption, and maintenance of Haibu Diabetes among patients, caregivers, and health care providers (HCPs).
- Aim 3: To describe the impact of Haibu Diabetes on health care improvement, including patient experience, HCP experience, and operational workflow.

### Hypotheses

For Aim 1, we hypothesize that using Haibu Diabetes will lead to improved clinical outcomes, as evidenced by a greater proportion of users who review glucose data since the last visit and adjust insulin dose since the last visit compared with nonusers; lower average HbA_1c_ among users compared with nonusers (an absolute decrease of 0.5%); and increased TIR among users compared with nonusers.

For Aim 2, we hypothesize that Haibu Diabetes will be feasible to implement, acceptable to both patients/families and HCPs (as measured by surveys, interviews, and engagement with the platform), and will demonstrate sustained use, defined as at least weekly use of the platform throughout the approximately 12-month study period.

For Aim 3, we hypothesize that patient/caregiver and HCP users will be satisfied with Haibu Diabetes and report improved care experiences, and that Haibu Diabetes will enhance perceived clinical and operational workflow efficiency.

## Methods

### Participants and Eligibility

Eligible HCPs will include endocrinologists, endocrinology trainees, diabetes nurse educators, and allied health professionals (such as registered dietitians and social workers) actively providing care to pediatric patients in the Diabetes Clinic at BC Children’s Hospital (BCCH). Patients will be eligible if they are under the care of a participating endocrinologist, are aged 3 to younger than 18 years, and have a confirmed diagnosis of T1D for at least 6 weeks prior to recruitment, with a minimum of 2 clinic appointments anticipated during the study period. Patients must use a CGM and either basal-bolus insulin injections or an insulin pump compatible with the current Haibu Diabetes integration via Glooko and Dexcom, so that device data can be displayed within Haibu Diabetes. In practice, eligible devices are expected to primarily include Dexcom and Omnipod, both of which are covered under BC’s public health system’s Fair Pharmacare program [20]. The primary caregiver (parent or legal guardian), defined as the individual primarily responsible for daily diabetes management, must feel comfortable communicating in English and be able to engage with study materials.

Patients will be excluded if they are in foster care (unless independently managing their diabetes and deemed eligible on a case-by-case basis), use non–Health Canada–approved do-it-yourself automated insulin delivery systems, or are undergoing active treatment for mental health conditions such as eating disorders or depression.

### Recruitment, Consent, and Assent

Following informational sessions led by the study team, HCPs will be recruited via invitation emails from the research assistant (RA). A clinic administrator will identify eligible patients and caregivers under the care of participating endocrinologists using a clinic database and distribute electronic study information letters on behalf of the investigators, including RA contact details and a link to a REDCap [21,22] eligibility screening form. Families will be able to indicate interest by either contacting the RA directly or completing the screening form, after which eligibility will be confirmed. In-person recruitment will also be used, with an RA approaching patients in the clinic. Interested families will complete the eligibility survey on an iPad or access it later via a flyer with study details and a QR code.

All e-consent and e-assent forms will be administered via REDCap. Consent from HCP participants will be obtained prior to recruiting their patient/caregiver participants. Caregivers will provide consent for their own participation and for their minor child. Children aged 7 to 17 years will additionally provide assent. For participants aged 17 years or older, capacity to consent will be formally assessed through consultation with HCPs prior to enrollment.

### Setting

This quasi-experimental trial will be conducted at a hospital that delivers multidisciplinary pediatric diabetes care in British Columbia, Canada. As BC’s only tertiary pediatric hospital, BCCH follows approximately 850 of the 2500 children in BC living with T1D, with the remainder followed at community-based clinics supported by local pediatricians and endocrinologists [23,24]. Participants will include children and adolescents living with T1D, their caregivers (ie, parents or legal guardians), and their HCPs involved in routine diabetes management.

This trial was registered on ClinicalTrials.gov on July 21, 2026 (NCT07712809).

### Intervention

#### Haibu Diabetes and Front-End Dashboards (Careteam)

##### Platform Overview and Architecture

Haibu Diabetes is an end-to-end solution comprising a front-end user interface (UI) and a backend core infrastructure that securely receives multiple data streams (ie, CGM, insulin pump, patient-reported, and clinical visit data). The front-end UI of Haibu Diabetes is powered by Careteam Technologies (Careteam) and is used by both patients/caregivers (“patient dashboard”) and HCPs (“HCP dashboard”), where they can view the same data and insights, and communicate and collaborate to support T1D care before, during, and between clinic visits.

##### Patient/Caregiver Dashboard

The patient dashboard was developed in response to HCP and patient/patient family preferences as measured by needs-assessment surveys [25]. Patients/caregivers access their patient dashboard using their smartphone or Careteam’s web-based app on their computer. During onboarding, patient and caregiver participants will have the option to meet with a study team member via a video call to guide them through setting up and registering an account on the platform for use during the trial. During this session, the study team member will assist participants with the following steps: accepting the invitation to join Careteam; registering for an account; reviewing and accepting the terms of use; adding multifactor authentication; understanding how to connect their diabetes devices; adding a primary supporter (if needed); and exploring the platform (Figure 1). Alternatively, caregiver and patient participants can opt to onboard independently using detailed onboarding support materials provided by the study team.

**Figure 1.**
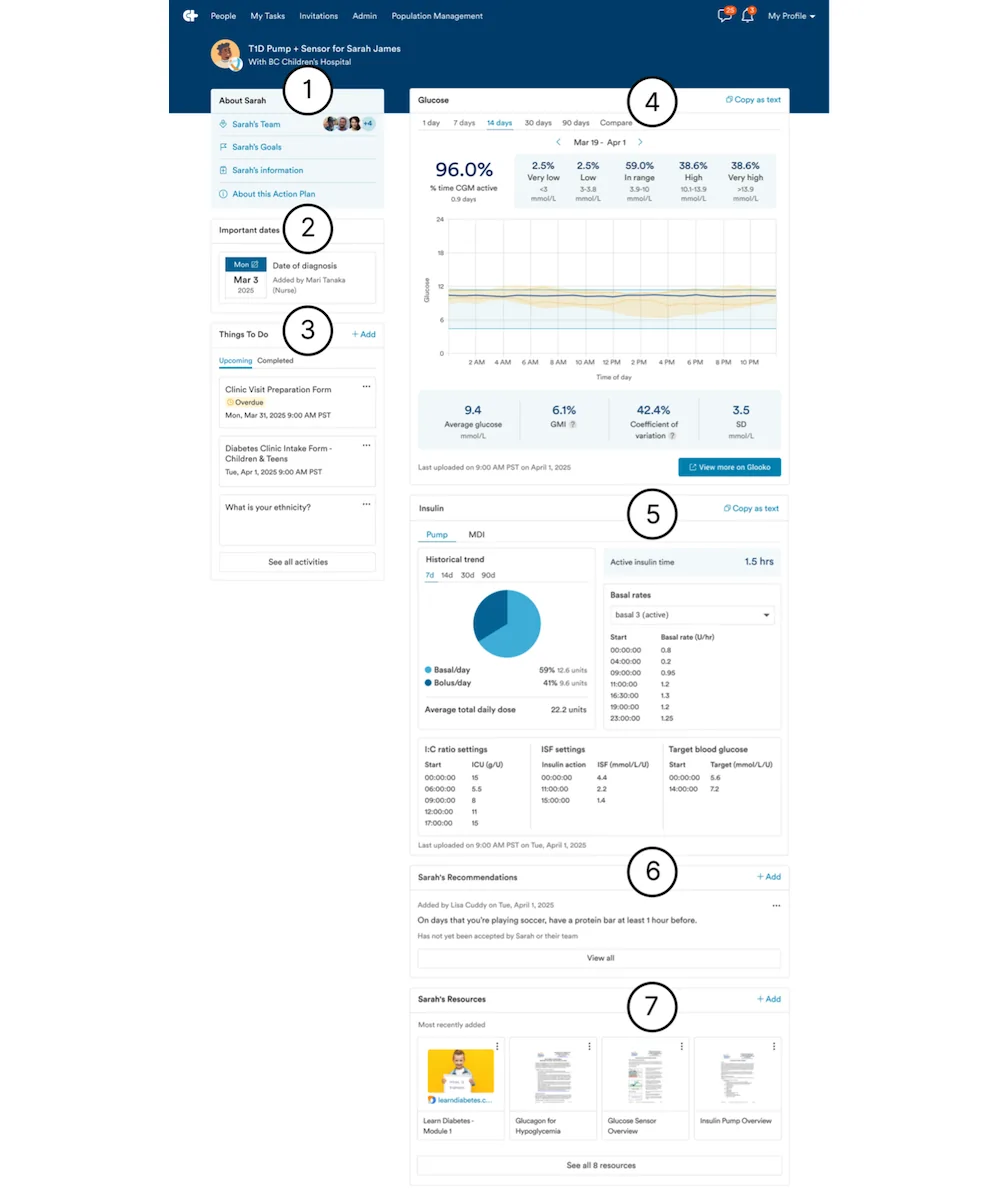
Features of the dashboard.

At any time after onboarding, patients/caregivers can connect their diabetes devices through Dexcom, a CGM platform [26], and Glooko [27], a diabetes device data aggregation platform, allowing CGM and pump data to automatically populate the dashboard for easy viewing. Additional dashboard features available to patients are manual entry of basal-bolus insulin doses and next appointment, and completion of forms/surveys (eg, clinic preparation form, transition readiness survey) or reminders/tasks (eg, complete blood work, upload pump). Patients/caregivers can also view recommendations from their HCP and navigate to commonly used educational resources.

Features are labeled numerically:

1. The About section contains patient information, including their team, goals, and other details.
2. The Important dates section contains the date of diagnosis.
3. The Things to do section contains forms assigned to the patient by the HCP.
4. The Glucose section displays data from the patient’s Glooko account or Dexcom once connected. Users can view the data in 1-, 7-, 14-, 30-, and 90-day formats and compare weeks. The summary below the graph shows average glucose, glucose management indicator, coefficient of variation, and SD. A button in the lower right links to Glooko for viewing the data there in more detail. A Copy as text button in the top right allows HCPs to copy this data into electronic medical record (EMR) notes.
5. The Insulin section has 2 view options depending on whether the patient uses an insulin pump or multiple daily injections (MDI). For pump users, data flow from their diabetes management device; for patients on MDI, information is entered manually.
6. The Recommendations feature allows HCP users to send recommendations, which patients and caregivers can accept or decline with a note to the HCP.
7. The Resources feature allows HCPs to send resources from a preset resource library or share a one-time resource when the library does not contain the needed resource.

##### HCP Dashboard

The HCP dashboard is web-based and accessed with the same credentials used for their health system apps (eg, EMRs) before, during, and between clinical encounters. HCPs can visualize and review patient data (CGM, insulin pump, responses to forms/surveys), review/edit basal-bolus insulin doses, assign tasks or forms/surveys to complete, and input their recommendations (ie, insulin dose adjustment). Copy-and-paste features allow HCPs to easily transfer information (eg, CGM metrics, pump settings, basal-bolus insulin doses) from the dashboard into EMR documentation. HCPs, through recommendations or tasks, can easily guide patients to resources and services, including clinic-approved internal and external resources and virtual educational modules [28].

### Haibu Diabetes Intelligent Core and Data Flows

#### Data Sources and Types

The intelligent back-end core of Haibu Diabetes is designed to accommodate external integrations, which currently are Glooko and the BC Pediatric Diabetes Registry (BC-PDR) [29]. These data are managed via 2 separate hubs for the storage of clinical (identifiable: Childhood Diabetes Data Hub [CDDH]) and research (deidentified: Childhood Diabetes Research Hub [CDRH]) data.

Data can be broadly sorted into 3 categories:

1. Patient- or HCP-inputted data: demographics (eg, ethnicity), forms or surveys completed by the patient, or recommendations inputted by the HCP during or between clinical visits.
2. Device data: data from CGMs and insulin pumps, ingested via external integrations (primarily Glooko and Dexcom at this stage).
3. Clinical data: patient- and visit-level data from clinical diabetes visits, including sociodemographic, anthropometric, laboratory results, technology use, and diabetes management behaviors (see Multimedia Appendix 1) from BC-PDR.

#### Clinical Hub (Identifiable): CDDH

The CDDH is where identifiable data from the 3 streams above are aggregated and can be used for individual patient care. For example, the CDDH enables visualization of device data flowing into the Haibu Diabetes back-end via Glooko and Dexcom for the patient and their HCP on the front-end UI. The CDDH has been built to accommodate more external integrations over time (eg, with other device companies, registries, or EMRs).

#### Research Hub (Deidentified): CDRH

Data from the CDDH are deidentified, transferred, and stored in a Structured Query Language data lake (a centralized database for structured data storage and querying) that comprises the CDRH, which holds deidentified data to form a data lake to be accessed by researchers and data scientists to run queries and develop and validate advanced analytics models (ie, machine learning). The data in the CDRH are curated to meet the requirements of the intended research: training machine learning algorithms to predict undesirable outcomes in pediatric diabetes. The CDRH will prospectively receive deidentified data from the CDDH on a scheduled basis (eg, weekly), depending on the source and the need.

### Study of the Intervention

The Intelligent Care Platform trial is a nonrandomized, quasi-experimental study evaluating the implementation and effectiveness of Haibu Diabetes in routine care at the BCCH Diabetes Clinic.

Participants will be enrolled in the intervention arm through recruitment and onboarding to Haibu Diabetes as part of routine care workflows. Comparative analyses will use a matched control group selected from the BC-PDR (see Multimedia Appendix 1 for the BC-PDR data dictionary). The registry includes patient-level information (eg, demographics, diabetes presentation) and clinical encounter data (eg, laboratory results, anthropometrics, behavioral measures, and diabetes management) drawn from EMRs.

Haibu Diabetes exposure, and therefore Haibu Diabetes users, will be defined a priori as: (1) account activation and (2) either (a) at least one data source connected (eg, CGM and/or pump upload) or (b) at least 1 meaningful platform interaction (eg, viewing data, completing a task/form, or messaging). Platform usage analytics will be used to quantify engagement (eg, logins, data views, tasks completed, messages sent). Nonusers of Haibu Diabetes will be extracted from the BC-PDR and will not have access to Haibu Diabetes during the study period.

The index date for intervention patients is the date of enrollment. As comparator patients have no such date, index dates will be assigned by sequential matching: each intervention patient will be matched to a comparator who is eligible, under observation, and event-free at that intervention patient’s index date, and the comparator inherits that index date.

### Study Procedures and Data Collection Schedule

Each participant in the intervention arm will be enrolled in the study for 12 months (48 wk). Data will be collected from patients, caregivers, and HCPs at baseline onboarding, after clinic visits, midstudy, and at the end of the study (Figure 2). At structured onboarding, before using Haibu Diabetes, participants will complete demographic and baseline assessments, after which they will be encouraged to use Haibu Diabetes for T1D management during and between routine clinical care encounters. Specifically, they will be advised to identify a consistent weekly time to use Haibu Diabetes to review glucose trends, assess patterns in their data, and make changes independently when they feel confident. When additional support is needed, participants will be instructed to contact the diabetes care team, including the diabetes nurse, using established communication pathways (eg, via email or phone). Tasks and check-ins will be programmed within Haibu Diabetes to support weekly data review, including prompts to reach out for help when needed. Detailed data collection procedures across 3 study phases are described below.

**Figure 2.**
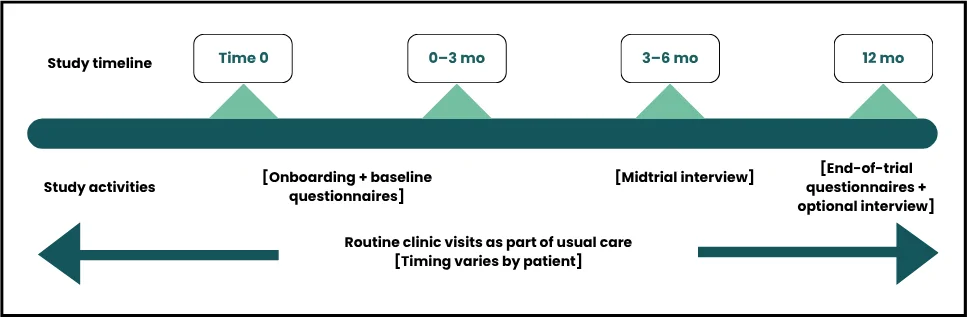
Study timeline.

Clinic visits occur as usual and may take place at variable times within each interval shown (0‐3, 3-6, and 6-12 mo).

Phase 1 (baseline, onboarding, and routine clinic visits) includes baseline questionnaires administered via REDCap, including the patient/caregiver demographic survey, the eHealth Literacy Scale [30], diabetes distress and health-related quality of life questionnaires (completed by the parent or patient depending on the measure), and baseline patient-centeredness [31-33], as well as a HCP demographic survey. Caregivers who decline participation will be invited to complete an optional refusal survey. Following onboarding, participants complete a post-onboarding survey that captures patient/caregiver perspectives on platform usability, security, and data sharing, including the System Usability Scale [34]. After routine clinic visits, patients/caregivers and HCPs complete postclinic surveys capturing their experience of using the platform during and between clinical encounters.

Phase 2 (midtrial) includes semistructured qualitative interviews with a purposive subsample of approximately 10 to 15 patient/caregiver participants to capture experiences after approximately 6 months of platform use, typically following the second clinic visit, depending on visit timing. Participants will be selected to capture variation in demographic and clinical characteristics. Interviews will explore barriers, facilitators, usability issues, and implementation considerations, including issues identified through the midtrial surveys. This sample size is expected to provide sufficient depth to identify key issues, with recruitment continuing until no major new themes are emerging.

Phase 3 (end of trial) will include end-of-study semistructured qualitative interviews with a purposive sample of approximately 15 to 20 patient/caregiver participants (which may include participants interviewed at midtrial) and 5 to 7 HCP participants. These interviews will explore barriers, facilitators, usability issues, and implementation-relevant experiences arising over the full trial period, including perceived impacts on care processes, communication, self-management support, and clinical workflow. Participants will again be selected to capture variation in relevant characteristics and experiences, with recruitment continuing until no major new themes are emerging. End-of-study surveys, including repeated patient-centeredness and Technology Acceptance Model [35] measures, will also be administered to all patient/caregiver and HCP respondents. Diabetes distress and health-related quality-of-life questionnaires will also be repeated. Routine clinical outcomes are collected through the BC-PDR at routine clinic visits as part of standard care (Table 1).

**Table 1.** Data collection tools and time points.

Data source	Onboarding (baseline)	Postroutine clinic visit	End of trial (12 mo)
HbA_1c_^a^, time in range, and diabetes self-care behaviors (2 yes/no items: “reviewed glucose data since last visit” and “changed insulin dose since last visit”)	**✓**		**✓**
Caregiver optional refusal survey^b^	**✓**		
Patient/caregiver demographic survey^b^	**✓**		
Patient-centeredness measure [31-33]^b^^,^^c^	**✓**		**✓**
HCP^d^ demographic survey^b^	**✓**		
Postonboarding survey^b^	**✓**		
System Usability Scale [34]^c^	**✓**		
Diabetes distress^c^ (measured using P-PAID-C^e^ [36], PAID-Peds^f^ [36], or PAID-PR^g^ [37,38])^h^			
Health-related quality of life^c^ (measured using T1DAL^i^) [39]	**✓**		
Postclinic patient/caregiver survey^b^		**✓**	
Postclinic HCP survey^b^		**✓**	
Patient/caregiver qualitative interviews (subset), conducted midtrial^b^			
Patient/caregiver/HCP qualitative interviews (subset)^b^			**✓**
End-of-study patient/caregiver survey (includes patient-centeredness)^b^			**✓**
TAM^j^ [35]^c^ (patient/caregiver)			**✓**
End-of-study HCP survey (includes TAM)^b^^,^^c^			**✓**

a HbA_1c_: hemoglobin A_1c_.

b A researcher-developed survey or interview guide.

c A validated survey.

d HCP: health care provider.

e P-PAID-C: Parent Problem Areas in Diabetes-Child.

f PAID-Peds: Problem Areas in Diabetes Survey-Pediatric Version.

g PAID-PR: Problem Areas in Diabetes Survey-Parent Revised Version.

h P-PAID-C and PAID-PR are parent-report measures; PAID-Peds is a youth self-report measure for ages 8–17 years; Parent versions include Parent of a Young Child (<8 years), Parent of Child (8-11 years), and Parent of Adolescent (12-17 years); all are parent-report measures of diabetes-specific health-related quality of life.

i T1DAL: type 1 diabetes and life.

j TAM: Technology Acceptance Model.

Throughout the trial, a structured researcher implementation log will be maintained from onboarding through trial close-out and used to document onboarding, training, workflow support, troubleshooting, and protocol deviations or adaptations.

### Measures

#### IRLM Logic Model and Framework Application

Outcomes and measures were specified a priori using the IRLM, which prespecifies the causal chain from context to implementation success by linking determinants, implementation strategies, mechanisms, and measurable outcomes [16]. Determinants were identified and prioritized using the Consolidated Framework for Implementation Research (CFIR) [40], which organizes multilevel determinants across domains such as intervention characteristics, inner setting, outer setting, characteristics of individuals, and process. Implementation strategies were selected and specified using the Expert Recommendations for Implementing Change (ERIC) taxonomy [41], a standardized compilation of implementation strategies. For each strategy, hypothesized mechanisms were articulated and mapped to prespecified outcomes spanning implementation, service, and clinical and patient domains. Implementation outcomes were guided by Proctor’s implementation outcomes framework [42] and organized using the RE-AIM (Reach, Effectiveness, Adoption, Implementation, Maintenance) framework [43] to structure assessment across implementation outcome domains. Service outcomes were aligned with the quintuple aims [44] of health care, and clinical/patient outcomes were aligned with T1D management goals.

Figure 3 presents the completed IRLM for the trial, mapping determinants to strategies, mechanisms, and outcomes. Table 1 summarizes the operationalization of outcomes through data collection tools, sources, and time points.

**Figure 3.**
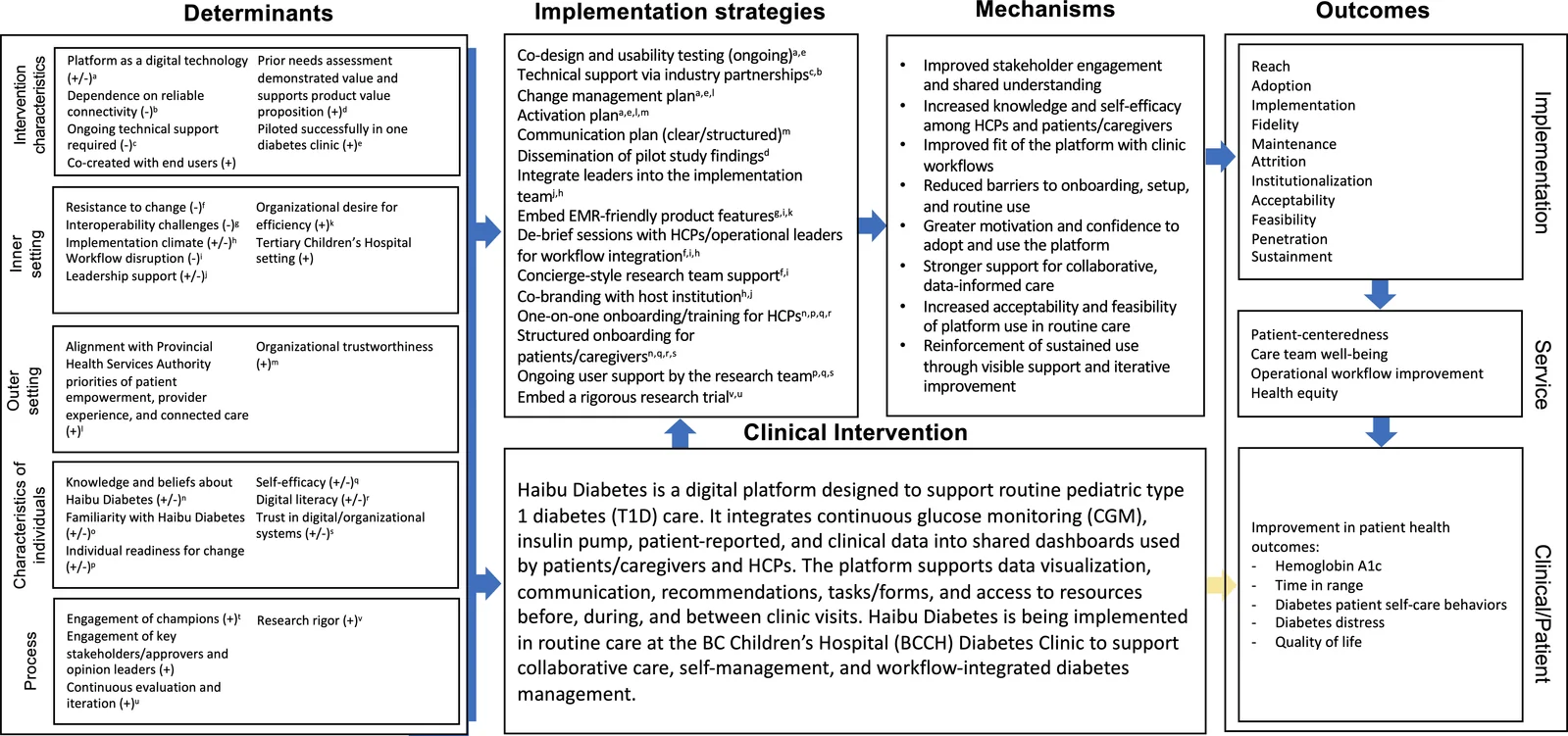
Implementation Research Logic Model for the Intelligent Care Platform trial. ± used to indicate whether each determinant is expected to function primarily as a facilitator or barrier. EMR: electronic medical record; HCP: health care provider.

Figure 3 presents the IRLM for the study in a left-to-right sequence. The first column (Determinants) summarizes contextual determinants identified and prioritized for this setting (CFIR-informed), organized across domains including intervention characteristics, inner setting, outer setting, characteristics of individuals, and process. The second column (Implementation Strategies) lists the implementation strategies selected to address the prioritized determinants (ERIC-specified). The third column (Mechanisms) summarizes the hypothesized processes through which the selected strategies are expected to influence determinants and support implementation. The fourth column (Outcomes) specifies the prespecified outcomes linked to these pathways, grouped as implementation outcomes (assessed across RE-AIM domains and Proctor-defined implementation outcomes), service outcomes (aligned with the quintuple aim domains), and clinical/patient outcomes (aligned with T1D management goals). The arrows indicate the hypothesized causal chain linking determinants to strategies, mechanisms, and outcomes. The “Clinical Intervention” box provides a concise description of Haibu Diabetes as the intervention being implemented and evaluated.

#### Implementation Outcomes

Reach: Assessed using the research consent log to track refusal rate, an optional refusal survey, and demographic surveys to characterize the eligible and approached population, quantify participation, and document reasons for nonparticipation.

Effectiveness: See clinical/patient outcomes below.

Adoption: Assessed using platform usage analytics to characterize engagement and continued use of Haibu Diabetes among intended users.

Implementation (including fidelity, feasibility, and workflow integration): Fidelity and feasibility will be assessed using the researcher’s implementation log. The log will capture timing, participant type, contact mode and trigger, implementation activities (eg, onboarding steps, training/education, workflow coordination, troubleshooting), resolution status and time to resolution, escalation, staff effort, and protocol deviations. Adaptations (what changed, when, by whom, and why) will be documented using the fields aligned with the Framework for Reporting Adaptations and Modifications-Expanded within the log [45]. Operational workflow integration will be assessed using qualitative interviews, meeting minutes from operational debrief sessions (with operational and divisional leadership), and EMR-derived indicators of clinic burden (counts/rates of urgent calls/emails and emergency department visits).

Maintenance (institutionalization): Assessed using standard operating procedures, onboarding and training materials to document routinization and integration of Haibu Diabetes-related practices within the clinic.

Study retention (attrition): Assessed using the research consent log, refusal survey, and demographic surveys.

In addition, Proctor-defined implementation outcomes are assessed as follows: acceptability using the Technology Acceptance Model [35] survey; feasibility using researcher implementation logs; penetration using the research consent log and demographic surveys; and sustainment using the research consent log, refusal survey, demographic surveys, and platform usage analytics.

#### Service Outcomes

Service outcomes assess health care improvement domains aligned with the quintuple aims and are operationalized using survey and qualitative methods. Patient/caregiver experience is measured using the validated Person-Centered Primary Care Measure [33-35] for both patients and caregivers and semistructured interviews.

#### Clinical/Patient Outcomes

Clinical/patient outcomes are collected during routine clinical encounters and are available through the BC-PDR (Multimedia Appendix 1). Outcomes include diabetes self-care habits and glycemic outcomes. The primary effectiveness outcome is HbA_1c_. Secondary effectiveness outcomes include TIR and diabetes self-care behavior items (eg, glucose data review and insulin dose adjustment where relevant).

### Analysis

Analyses will integrate quantitative and qualitative methods to evaluate clinical and implementation outcomes, including comparative analyses between Haibu Diabetes users and matched nonusers, as well as thematic analysis of qualitative interview data.

Quantitative: Both descriptive and comparative statistical methods will be used to evaluate clinical outcomes between Haibu Diabetes users and nonusers. Descriptive statistics, including means, SDs, medians, and proportions, will summarize baseline characteristics, key metrics such as platform reach, implementation fidelity, and usage patterns, and patient/caregiver-reported outcomes (eg, satisfaction, perceived ease of use, and patient-centeredness).

To determine the effectiveness of Haibu Diabetes, we will conduct propensity score matching (matched on patient age, patient and caregiver gender, baseline HbA_1c_, TIR, and insulin regimen [ie, MDI vs insulin pump]) to select patients from the BC-PDR (nonusers of Haibu Diabetes) who are as close as possible to those in the study (Haibu Diabetes users). The primary estimand is the average treatment effect in the treated, with the average treatment effect also estimated.

Intervention patients will be matched 1:1 to comparator patients without replacement. The matching algorithm will be selected from a prespecified sequence, escalating only if the balance criteria below are not met: (1) greedy nearest-neighbor matching with a caliper of 0.2 SDs of the logit propensity score; (2) optimal matching with the same caliper; (3) caliper narrowing to 0.1 SD; and (4) full matching. All algorithm selection will be performed with outcome data masked, on the basis of covariate balance between intervention and comparator groups. Analyses will be restricted to the region of common support, defined by the overlap region of the propensity score distributions (patients with scores outside the range common to both groups will be excluded), together with the caliper restriction above. Balance will be assessed by standardized mean differences for all matching covariates, their squares, and prespecified pairwise interactions, with a standardized mean difference below 0.10 taken as adequate balance. Unmatched participants will be excluded from the primary analysis and compared descriptively to those included.

Regression analyses of the matched sample will include covariate adjustment to provide “doubly robust” estimates. CIs will be computed via the nonparametric bootstrap with 1000 resamples. Secondary analyses may include using all nonusers of Haibu Diabetes from the BC-PDR as a control group, with regression adjustment for the above confounders. In sensitivity analyses retaining the full cohort, the treatment effect will be reestimated using inverse probability weighting. All analyses will be conducted using R statistical software (version 4.4.4) [46]. Outcomes for which comparative analyses will be conducted include diabetes self-care habits and glycemic control (HbA_1c_ levels and TIR).

Missing data will be summarized by outcome and time point. For matched analyses, the primary approach will use available outcome data as recorded in routine care. Sensitivity analyses will include multiple imputation [47,48].

Qualitative: Interviews will be transcribed verbatim using transcripts and audio recordings generated via Zoom’s recording capabilities and validated by the research team for accuracy prior to analysis. Using NVivo, 2 members of the research team will individually open-code 4 different interviews each. They will then meet to discuss and decide on a coding system along with definitions for each code using Braun and Clarke’s thematic analysis methodology [49]. The first 4 interviews will be independently coded by 2 team members using the agreed coding framework and definitions. The coders will meet to compare coding, discuss discrepancies, and refine the coding framework as needed. The remaining interviews will then be coded using the finalized framework, with any coding uncertainties discussed and resolved through regular team meetings. Once coding is complete, coded excerpts will be exported from NVivo and reviewed across the dataset to identify emerging themes. The research team will meet to discuss and refine themes and subthemes, resolve interpretive differences, and select exemplar quotations. Interview participants may be invited to review emerging themes and comment on whether the interpretations reflect their experiences.

### Sample Size

The primary effectiveness outcome for power calculations is HbA_1c_. The sample size was calculated to detect a clinically meaningful absolute decrease of 0.5% in HbA_1c_ levels between Haibu Diabetes users and matched controls, assuming a SD of 1.0 percentage points. This assumption is broadly consistent with prior T1D trials reporting HbA_1c_ SDs of approximately 1.2 to 1.4 percentage points [50]. Using a 2-sided paired *t* test with α=.05 and 80% power, 64 participants per group were required; allowing for 20% attrition, the final target sample size is 80 participants per group (total N=160, with 80 recruited). Recruitment will aim to achieve representation of 25% of youth aged 15 to 17 years who are self-managing diabetes and 75% of participants with a parent or guardian as the primary caregiver, a balance of 50% male and 50% female participants, and an ethnic distribution reflective of T1D prevalence in Canada (~70% Caucasian, 15% South Asian, and 15% other) through targeted recruitment.

### Ethical Considerations

This study was approved by the University of British Columbia’s Children and Women’s Research Ethics Board (H25-00649; approval date: July 16, 2025) and conducted in accordance with the Tri-Council Policy Statement 2 and the Declaration of Helsinki. Electronic informed consent will be obtained from participating HCPs and caregivers, with caregiver permission for minor participants and assent obtained from children aged 7 to 17 years. Participants will be offered an honorarium of CAD $5-$20 (US $3.53-$14.10; CAD $1=US $0.70502 as of October 15, 2026), depending on survey length, in the form of a gift card for each completed survey, and CAD $60 (US $42.30) for each completed interview. HCP participants will be offered a CAD $5 (US $3.53) gift card for each completed survey and CAD $60 (US $42.30) for each completed interview.

Harms: Anticipated risks include perceived burden associated with onboarding or ongoing platform use, distress from viewing health data, and privacy or confidentiality concerns. Participants may discontinue use of Haibu Diabetes at any time without affecting clinical care. Any privacy incidents, participant complaints, or unintended consequences identified during the study will be documented and, as required, escalated to the principal investigator (SA) and the institutional privacy and ethics bodies. For participant distress, appropriate referral pathways to clinic-based social workers or other mental health supports will be established.

Monitoring and Auditing: A formal independent data monitoring committee is not planned because this is a minimal-risk, nonrandomized digital platform evaluation embedded in routine care. The study team will conduct internal monitoring focused on recruitment pace, data completeness, and protocol adherence. Any protocol deviations will be logged and reviewed by the principal investigator (SA).

### Data Management

Study survey data will be collected and stored in REDCap. Platform usage analytics and Haibu Diabetes data streams will be stored within the Haibu Diabetes infrastructure, as described (CDDH/CDRH). Registry outcomes will be obtained from the BC-PDR. Data quality will be supported through standardized operating procedures, routine completeness checks, and documentation of data extraction and transformation steps to ensure reproducibility in analysis. Deidentified analytic datasets will be stored on secure institutional servers, with access restricted to authorized study personnel.

Protocol Amendments: Ancillary and posttrial care: Participants will continue to receive standard clinical care regardless of study participation or Haibu Diabetes use. No additional clinical services are provided beyond usual care as part of this protocol, and any clinical issues identified during participation will be managed through routine clinical pathways.

Dissemination: Findings will be disseminated through peer-reviewed publications and conference presentations. Where feasible, study summaries will be shared with participating families and clinic stakeholders in accessible formats. Authorship will follow established journal and institutional guidance.

## Results

Study funding commenced in January 2023, and research ethics approval was obtained in July 2025. Patient participant recruitment began in September 2026. As of October 2026, 7 participants had consented to participate in the study. Data collection is expected to continue through December 2027, with data analysis to follow. Study findings are anticipated to be published in summer 2028.

## Discussion

### Summary

This protocol describes a type 2 hybrid implementation-effectiveness evaluation of Haibu Diabetes embedded in routine pediatric T1D care at BC Children’s Hospital in Vancouver, Canada. Building on a pilot of an earlier version of the platform that demonstrated feasibility and potential benefit and identified opportunities for refinement, this study evaluates the next-generation Haibu Diabetes and the conditions needed for its sustained use in routine care [17]. By combining clinical effectiveness analyses with an implementation evaluation guided by the IRLM, the study is designed to address two practical questions: whether Haibu Diabetes improves outcomes in real-world practice and what determinants and strategies shape adoption, integration, and sustainment within clinic workflows [18].

### Interpretation

The implementation evaluation is designed to explain how Haibu Diabetes fits within routine clinical workflows and where breakdowns may occur. By combining usage analytics, implementation logs, surveys, interviews, and operational indicators, we can interpret outcomes in context and separate two common explanations for limited effects. One possibility is intervention failure, in which Haibu Diabetes does not improve outcomes even when used as intended. Another possibility is implementation failure, where Haibu Diabetes could be beneficial but uptake, fidelity, or workflow integration is insufficient to achieve impact [51,52].

We will also examine whether the mechanisms targeted by Haibu Diabetes are reflected in real-world use [53,54]. For example, if Haibu Diabetes reduces previsit preparation burden or enables more timely therapeutic adjustments, these changes should be observable in usage patterns and stakeholder experiences. Linking hypothesized mechanisms to measured engagement and workflow integration may help identify which platform features and implementation conditions are most important for clinical benefit.

If Haibu Diabetes shows benefit and can be implemented feasibly in routine care, the results can guide broader adoption by clarifying what clinic-level supports are needed, such as training, structured onboarding, workflow redesign, and clear team roles. These supports are often essential for sustainment because programs can lose momentum when ongoing operational support is lacking [55].

If effects are modest or vary across users, the IRLM-informed evaluation will be useful in identifying where the implementation pathway breaks down (eg, limited device integration, documentation burden, or misalignment with visit timing). This information can guide targeted adaptations before wider dissemination, if appropriate.

Beyond informing scale-up, study findings may also support iterative refinement of both the Haibu Diabetes platform and its implementation approach. For example, barriers related to usability, data integration, or workflow fit could inform changes to platform features, onboarding processes, training, and team workflows. These findings may also be used to refine the IRLM by updating key determinants, mechanisms, and implementation strategies based on empirical experience from routine care.

### Strengths

A key strength of this study is the explicit, theory-informed specification of the implementation pathway. The IRLM links determinants, implementation strategies, hypothesized mechanisms, and outcomes in a single coherent structure, which strengthens transparency and supports interpretation beyond a simple “worked/didn’t work” conclusion [16]. By making assumptions and evaluation targets explicit, the IRLM also improves reproducibility and helps explain how and why Haibu Diabetes is expected to be adopted and integrated into routine care.

Consistent with IRLM guidance, we used complementary implementation frameworks to specify each element of the logic model and support standardized reporting. CFIR [40] informed the identification and description of multilevel determinants (barriers and facilitators), the ERIC taxonomy [41] informed the specification of implementation strategies, and Proctor’s implementation outcomes framework [42] informed the definition of key implementation outcomes. We organized these outcomes using RE-AIM [43] to ensure evaluation extends beyond clinical effectiveness to include reach, adoption, implementation, and maintenance [56]. Taken together, this approach increases the interpretability and comparability of findings with those from other digital health implementation studies.

A further strength is the inclusion of service outcomes aligned with the quintuple aims [44] of health care improvement (patient experience, population health, and care team well-being). This broader framing supports assessment of whether Haibu Diabetes meaningfully improves care delivery and workflow, not only glycemic outcomes.

Finally, the quasi-experimental matched-control design using routinely collected registry data aligns with the goal of evaluating Haibu Diabetes under real clinical conditions. This pragmatic approach increases external validity and reduces participant burden by leveraging outcomes captured during usual care, rather than requiring additional study visits or intensive primary data collection [57-60].

### Limitations

Nonrandomized allocation introduces a risk of residual confounding [61]; for instance, families who adopt Haibu Diabetes may differ from nonusers in motivation, resources, or baseline engagement. Propensity score matching and covariate adjustment are planned to reduce these differences, and we will report balance diagnostics to assess the performance of matching. However, even with these methods and doubly robust estimation, the results should be interpreted as comparative effectiveness estimates rather than definitive causal effects. Potential contamination is an additional limitation because Haibu Diabetes users and matched control patients may receive care from the same clinical team. HCP experience with Haibu Diabetes could influence care provided to nonusers, thereby reducing differences between intervention and matched control care. This contamination cannot be directly accounted for in the planned analyses and could attenuate observed between-group differences.

There are also limitations in deriving outcome measures from routine care documentation in the EMR. Outcomes derived from routine care may be measured inconsistently, as documentation practices vary and missing data are common in EMR- and registry-based research [62-64]. Self-care items recorded in the registry may vary across visits and providers because documentation practices differ. Clear reporting of missingness patterns and sensitivity analyses will therefore be important, particularly if data completeness differs across patient subgroups.

A further limitation is that the matched control group drawn from the BC-PDR will have only clinical data. Diabetes distress measures (PAID-C [36], PAID-Peds [36], or PAID-PR [37,38]) and quality of life (T1DAL [39]) are not assessed using validated tools as part of standard clinical care and will therefore only be collected from Haibu Diabetes users.

This evaluation is also conducted at a single site. As a result, the findings may not fully generalize to other pediatric diabetes clinics with different staffing models, workflows, or patient populations. Additional multisite studies will be needed to assess transportability and to understand how local context influences implementation and outcomes.

### Equity Considerations

Equity is a core consideration in digital health because barriers related to access, language and cultural fit, and digital health literacy can shape who benefits from technology-enabled care [65-68]. Measuring digital health literacy and documenting reasons for nonparticipation can help assess whether Haibu Diabetes risks widening existing gaps (eg, if uptake is higher among families with greater resources or fewer structural barriers) [68-70]. To support equitable scale-up, we will report reach and adoption stratified by key sociodemographic characteristics to identify differential uptake and inform targeted implementation supports [71-73].

### Future Directions

Future work should evaluate whether findings from this single-site study carry over to other pediatric diabetes clinics with different staffing models, workflows, and patient populations. This includes assessing longer-term sustainment after research supports (eg, onboarding assistance and study-related monitoring) are reduced or withdrawn, because sustainment often depends on ongoing organizational supports and context.

Future studies should also examine whether effects differ across participant subgroups (eg, by age, insulin regimen, baseline glycemic control, or digital health literacy). Understanding heterogeneity can clarify who benefits most and can guide more targeted implementation approaches.

Finally, it will be important to determine what level of Haibu Diabetes engagement is associated with clinically meaningful improvement and what workflow conditions support that engagement. Establishing engagement “thresholds” and required workflow elements can inform platform optimization and more practical implementation guidance for scale-up.

### Conclusions

Overall, this protocol is designed to generate both clinical outcome evidence and practical “how-to” knowledge needed to support real-world digital transformation in pediatric diabetes care. By evaluating effectiveness alongside implementation processes, the study will help determine not only whether Haibu Diabetes improves outcomes but also how it can be adopted, integrated, and sustained within routine clinic workflows. This combined approach addresses a common gap in digital health research, where evaluations may focus on clinical endpoints or usability without providing sufficient guidance for successful real-world implementation and scale-up.

## Supplementary material

10.2196/107634 Multimedia Appendix 1 British Columbia Pediatric Diabetes Registry: patient-level data.

10.2196/107634 Checklist 1 SQUIRE 2.0 checklist.

10.2196/107634 Peer Review Report 1 Peer review report from CIHR-JDRF Type 1 Diabetes and Precision Medicine Review Committee, Canadian Institutes of Health Research (CIHR).

## Abbreviations

BC-PDR: BC Pediatric Diabetes Registry
BCCH: BC Children’s Hospital
CDDH: Childhood Diabetes Data Hub
CDRH: Childhood Diabetes Research Hub
CFIR: Consolidated Framework for Implementation Research
CGM: continuous glucose monitoring
EMR: electronic medical record
ERIC: Expert Recommendations for Implementing Change
HbA_1c_: hemoglobin A_1c_
HCP: health care provider
IRLM: Implementation Research Logic Model
MDI: multiple daily injections
RA: research assistant
RE-AIM: Reach, Effectiveness, Adoption, Implementation, Maintenance
SQUIRE: Standards for Quality Improvement Reporting Excellence
T1D: type 1 diabetes
TIR: time in range
UI: user interface
