# Supplementary material for: Real-World Implementation and Evaluation of a Digital Health Platform for Children Living With Type 1 Diabetes: Protocol for a Real-World Implementation Study
Source: JMIR Res Protoc. 2026 Oct 30;15:e107634. doi: 10.2196/107634 (PMC13722103; doi:10.2196/107634)
Supplement: Supplementary file 1 [file resprot-v15-e107634-s003.pdf]

Review Type/Type d'évaluation: Committee Member 1/Membre de comité 1
Name of Applicant/Nom du chercheur: Amed, Shazhan
Application No./Numéro de demande: 485828
Agency/Agence: CIHR/IRSC
Competition/Concours: 2022-08-23 Team Grant: CIHR-JDRF Type 1 Diabetes and Precision Medicine/Subvention d'équipe : IRSC-FRDJ : Médecine de précision dans le diabète de type 1
Committee/Comité: Team Grant: CIHR-JDRF Type 1 Diabetes and Precision Medicine/Subvention d'équipe : IRSC-FRDJ : Médecine de précision dans le diabète de type 1
Title/Titre: Precise Treatment for Pediatric Diabetes: Providing the right care, for the right patient, at the right time, over time

---

Assessment/Évaluation:

Research Proposal

This a complex densely written application over 4 years for $875000
The applicants point out that management of childhood diabetes remains far from ideal and that glycemic outcomes often fail to reach target. Importantly they highlight that adverse psychological outcomes and DKA occur frequently, particularly in the adolescent age group.

They point out that data from glucose sensors and pumps are producing far more data than in the past and that these and other data are not collected systematically in one place and this makes it difficult for families and patients to use. They argue that providing data in one location using technology will enable families and patients to use the information to manage their diabetes more effectively and improve outcomes

They have developed a digital tool, TrustSphere which allows patients and fanily to access important diabetes information, like blood sugars and insulin doses streaming from glucose sensors and insulin pumps (smart insulin pens?). The technology also allows input from from the diabetes team, giving advice on management.

A pilot study is currently underway in 50 families measuring feasibliy and effects on HbA1c and other metrics (but which will not report until March 2023). In previous work the team has also developed a diabetes register, digital online platform and psychosocial support

In support of their application they cite other data systems which have adopted algorithms to predict outcomes such as those with rising glucose values. They will adapt these algorithms to target those in need of support who they hypothesise are likely to benefit from their intervention.

The work consists of 3 phases.

In phase 1 they will refine the TrustSphere digital tool.

Phase 2 – They will conduct a pragmatic trial in a larger sample of patients/families (N=150) and will generate a TrustSphere dataset that we will be able to compare between TrustSphere users and non-users, glycaemic outcomes and measures of and diabetes self-management (presumably insulin use and pump delivery. There is no mention of other important data such as hypoglycaemia, CHO intake or physical activity although I assume this is possible as well as ‘behaviours’ (I assume relate to self management.

**Review Type/Type d'évaluation:**	Committee Member 1/Membre de comité 1
**Name of Applicant/Nom du chercheur:**	Amed, Shazhan
**Application No./Numéro de demande:**	485828
**Agency/Agence:**	CIHR/IRSC
**Competition/Concours:**	2022-08-23 Team Grant: CIHR-JDRF Type 1 Diabetes and Precision Medicine/Subvention d'équipe : IRSC-FRDJ : Médecine de précision dans le diabète de type 1
**Committee/Comité:**	Team Grant: CIHR-JDRF Type 1 Diabetes and Precision Medicine/Subvention d'équipe : IRSC-FRDJ : Médecine de précision dans le diabète de type 1
**Title/Titre:**	Precise Treatment for Pediatric Diabetes: Providing the right care, for the right patient, at the right time, over time

Assessment/Évaluation:

They hope to identify and describe sub-populations of children living with T1D, at risk for what they labelsub-optimal outcomes, and heterogeneity in their lifestyle and self-management behaviours. How

Phase 3 – Design and Implement Precise Care:

In the final phase they will apply the validated predictive algorithms to the linked, multi-source dataset to predict patients at greatest risk and using a co-design approach with young people themselves and their families design interventions, presumably educational to to improve outcomes using a variety of virtual and digital approaches.

The strengths of this proposal are as follows
1) Work which builds on important previous initiatives and which targets those patients and families most at risk

2) Explores the potential of modern methods of data collection which clearly have potential to improve self care.

3) Using co-design provide a crucial contribution from patients and families themselves who have to live with the challenges of diabetes self management during one of the most difficult phases of their lives. (Parents having to transition care from management they are in charge to young people themselves). Adolescence is a time when many reject the burden of self management leading to a high risk of DKA and high glucose levels. It appears that the team have sufficient experience and expertise in user centred design to conduct this phase successfully.

However I have a number of important concerns.

1) There is an assumption to some extent that the mere provision of more data in an accessible way will facilitate more effective self- management. While I agree that these data have the potential to result in this desirable outcome it still requires active engagement and self-management, and frequently changes in behaviour. This is challenging to achieve particularly in teenagers who hate their diabetes and often refuse to access data or to act on it for many different reasons. As far as I can see there are no preliminary data provided and we will not know the result of the current pilot work until next year. What happens if the pilot fails to show feasibility or benefit? This is not raised as a potential risk, and the applicants fail to explain

Review Type/Type d'évaluation: Committee Member 1/Membre de comité 1
Name of Applicant/Nom du chercheur: Amed, Shazhan
Application No./Numéro de demande: 485828
Agency/Agence: CIHR/IRSC
Competition/Concours: 2022-08-23 Team Grant: CIHR-JDRF Type 1 Diabetes and Precision Medicine/Subvention d'équipe : IRSC-FRDJ : Médecine de précision dans le diabète de type 1
Committee/Comité: Team Grant: CIHR-JDRF Type 1 Diabetes and Precision Medicine/Subvention d'équipe : IRSC-FRDJ : Médecine de précision dans le diabète de type 1
Title/Titre: Precise Treatment for Pediatric Diabetes: Providing the right care, for the right patient, at the right time, over time

---

Assessment/Évaluation:

how they will address this.

2) In using real world data, I assume that the pilot and expanded trial are not comparing randomised patients and families, surely there is a significant risk of bias as those who already engage and self manage effectively are more likely to participate.

3) The applicants have not addressed the importance of including the science of behavioural change which seems to me, fundamental. Merely providing data and even advice is insufficient if it is not acted on.

4) How will applicants ensure the participation of those most at risk, often those from poorly served communities, those with social issues or those who often fail to engage. I would argue that additional effort and strategies need to be in place to try and ensure their participation as those in this category are particularly challenging to engage in activities such as co-design.

5) This is an expensive application and the applicants are requesting substantial funding for 4 years . In my country, in the panels I am involved in, we would expect applicants to provide clear and convincing stop/go criteria to demonstrate they are making appropriate progress at important stages. This allows the funding to be released in stages as the project progresses and reassures funders that public money is being used appropriately. These have not been provided in this application.

#2

•Originality of the Proposal

Some aspects of this application are original and interesting. The data collection platform seems advanced and there is every reason to expect it to present the information in an easy to read form. Co-design is being used more frequently in the diabetes field and has the potential to engage those who find it challenging to engage in self care. I am aware of applications elsewhere in the world who have used the approach to develop interventions that support adolescents by using co-design both to engage this age group and to try and ensure that the resulting intervention is fit for purpose.

#3

•Applicants and Team

**Review Type/Type d'évaluation:**	Committee Member 1/Membre de comité 1
**Name of Applicant/Nom du chercheur:**	Amed, Shazhan
**Application No./Numéro de demande:**	485828
**Agency/Agence:**	CIHR/IRSC
**Competition/Concours:**	2022-08-23 Team Grant: CIHR-JDRF Type 1 Diabetes and Precision Medicine/Subvention d'équipe : IRSC-FRDJ : Médecine de précision dans le diabète de type 1
**Committee/Comité:**	Team Grant: CIHR-JDRF Type 1 Diabetes and Precision Medicine/Subvention d'équipe : IRSC-FRDJ : Médecine de précision dans le diabète de type 1
**Title/Titre:**	Precise Treatment for Pediatric Diabetes: Providing the right care, for the right patient, at the right time, over time

Assessment/Évaluation:

This is generally a strong team, notably with clinical and psychology input; the expertise in data collection, handling and technology is also a strength. The applicants have a reasonably strong track record. The absence of a behavioural change scientist is a weakness.

#4

•Environment for the Research
The environment of the BC Children's Hospital seems pretty strong particularly in view of the significant initiatives the applicants have implemented over the last few years. The involvement of the Kansas City Hospital might be considered a further strength. No concerns.

#5

•Impact of the Research
The sub-optimal outcomes of young people with type 1 diabetes including mental health issues emphasises the importance of new approaches in supporting them and their families to achieve more effective self-management. This project has the potential to use new methods of data collection and presentation to be integrated into a package supporting patients and families in diabetes managements. But funding this expensive proposal in advance of the completion of the pilot/feasibility work seems premature. I have lowered my score accordingly.

Review Type/Type d'évaluation: Committee Member 2/Membre de comité 2
Name of Applicant/Nom du chercheur: Amed, Shazhan
Application No./Numéro de demande: 485828
Agency/Agence: CIHR/IRSC
Competition/Concours: 2022-08-23 Team Grant: CIHR-JDRF Type 1 Diabetes and Precision Medicine/Subvention d'équipe : IRSC-FRDJ : Médecine de précision dans le diabète de type 1
Committee/Comité: Team Grant: CIHR-JDRF Type 1 Diabetes and Precision Medicine/Subvention d'équipe : IRSC-FRDJ : Médecine de précision dans le diabète de type 1
Title/Titre: Precise Treatment for Pediatric Diabetes: Providing the right care, for the right patient, at the right time, over time

---

Assessment/Évaluation:

1. **Research Question**

The objective of the proposal stated by the applicant is to 'further develop TrustSphere and Carehub to support *patient-centered, data-driven, collaborative and personalized care* and the registry goal is to provide a new pool of patient-generated health data that can provide insight into the *heterogeneity in outcomes across different sub-populations* and which therapeutic strategies will work for whom.' The researchers intend to leverage their prior work and upcoming work to 'generate knowledge regarding the heterogeneity of the lived T1D experience and subsequent outcomes that will enable and inform new precision medicine approaches for the management of T1D in children and youth'.
This goal is consistent with the research areas of the funding opportunity in developing an approach to personalized care. However, the main focus of the application appears to be the development of applications and tools that may be used to improve care even if this proposal does not by itself provide a greater understanding of how individual characteristics can lead to better individualized diabetes management.

2. **Research Approach**

The research will use a novel digital health application called TrustSphere that integrates patient generated data from devices and self-report. It is proposed that these data will be used to determine individualized diabetes management approaches to improve outcomes in youth. TrustSphere is part of CareHub which provides a shared experience among patients, families, and providers. There also is a research portal where data can be shared in a registry.
A pilot study with separate funding will precede TrustSphere's feasibility in 50 patients/families with T1D and their healthcare providers. User feedback will be integrated into a second version of TrustSphere.
The proposal has 3 phases:
Phase 1 will expand the data capabilities of TrustSphere via integration with: (i) a broader group of diabetes device brands; (ii) the REDCap platform to collect patient reported outcome and experience measures (PROM/PREM); and (iii) a commercially available physical activity tracker.
Phase 2will include a pragmatic 'real-world' trial in which 150 patients/families will use TrustSphere at the BCCH Diabetes Clinic including the 50 from phase 1). Additionally, the expanded dataset in the TrustSphere Registry will be linked to the BC-Pediatric Diabetes (clinical) Registry (BC-PDR) to better understand characteristics and outcomes in sub-populations of children living with T1D to inform the design of personalized care pathways (Phase 3).
Phase 3will apply and validate existing algorithms that identify and predict undesirable outcomes (rising A1C, worsening time in range (TIR)) using the expanded TrustSphere Registry linked to the BC-PDR. In partnership with patients, families and pediatric healthcare providers (HCP), as well as industry partners, the CareHub and its dashboard will be further developed to effectively share insights generated via TrustSphere's expanded data capabilities in order to inform the personalization of care.

Review Type/Type d'évaluation: Committee Member 2/Membre de comité 2
Name of Applicant/Nom du chercheur: Amed, Shazhan
Application No./Numéro de demande: 485828
Agency/Agence: CIHR/IRSC
Competition/Concours: 2022-08-23 Team Grant: CIHR-JDRF Type 1 Diabetes and Precision Medicine/Subvention d'équipe : IRSC-FRDJ : Médecine de précision dans le diabète de type 1
Committee/Comité: Team Grant: CIHR-JDRF Type 1 Diabetes and Precision Medicine/Subvention d'équipe : IRSC-FRDJ : Médecine de précision dans le diabète de type 1
Title/Titre: Precise Treatment for Pediatric Diabetes: Providing the right care, for the right patient, at the right time, over time

---

Assessment/Évaluation:

The staged approached planned for the 3 phases is well described. The researchers should be able to recruit participants in a timely fashion to maintain the project timeline.

Phase 1

Integration with new devices such as activity tracker wearable may be challenging and may not be readily achieved due to lack of cooperation from companies or other factors. Even if accomplished, the specific wearable may be in use by only a small proportion of the intended patient population. While such integration has merit, the researchers should consider the ramifications to the algorithm development when such data are not available.

The plan to integrate with additional pumps and CGMs has merit but there are not details of how this will be accomplished and whether the companies have already been approached to determine their willingness to cooperate. The proposal does identify these potential risks but there may not be realistic mitigation strategies.

Phase 2

The aim in Phase 2 addresses 4 research questions. The first relates to whether TrustSphere is effective in improving diabetes self-management behaviors and outcomes. The second and third deal with heterogeneity related to patient-level or system level factors. The fourth relates to the feasibility of TrustSphere.

A critical aspect of the TrustSphere effectiveness evaluation will be the selection of the 190 patients/families. A clear recruitment strategy will be important to try to have an unbiased selection and it will be important to document information about those who are approached and decline. For the evaluation of effectiveness, the comparison group will be drawn from the BC-PDR registry through propensity score matching. The analytic approach has been well thought out.

There is concern that many patients and families will find that the burden of the patient-generated data collection exceeds their perceived benefit and consequently, data collection will be incomplete or hampered by a high dropout rate. HCPs also may find the requirements for their role to be burdensome It will be important that this is critically assessed after completion of the pilot study and also within this proposed study after version 2 is developed and that consideration be given to the burden on patients/families and providers outside the confines of this study once the processes would be implemented broadly.

With respect to the identification of heterogeneity of effect and relating to patient/family and system factors, much is already known and it seems unlikely that this proposal will uncover new information in this regard.

Phase 3

This phase will be applying algorithms developed by PA Clements at Childrens-Mercy Hospital in Kansas City to the researchers dataset to see how well it predicts patients at risk for rising A1C/worsening TIR. This seems like a reasonable aim and the dataset is likely to be useful in this regard. However, it seems unlikely that with a sample of 50 cases that the objective to include attributes not accounted for in the CMKC model will be useful.

An additional aim is to create in conjunction with stakeholders (patients/families, HCPs, and operational leaders), personalized care pathways to prevent undesirable outcomes based on the use of predictive algorithms. The proposal indicates that with patients, families and HCPs, the researchers will (i) co-create strategies to intervene in order to translate these data-informed insights into action, using TrustSphere as a

Review Type/Type d'évaluation: Committee Member 2/Membre de comité 2
Name of Applicant/Nom du chercheur: Amed, Shazhan
Application No./Numéro de demande: 485828
Agency/Agence: CIHR/IRSC
Competition/Concours: 2022-08-23 Team Grant: CIHR-JDRF Type 1 Diabetes and Precision Medicine/Subvention d'équipe : IRSC-FRDJ : Médecine de précision dans le diabète de type 1
Committee/Comité: Team Grant: CIHR-JDRF Type 1 Diabetes and Precision Medicine/Subvention d'équipe : IRSC-FRDJ : Médecine de précision dans le diabète de type 1
Title/Titre: Precise Treatment for Pediatric Diabetes: Providing the right care, for the right patient, at the right time, over time

Assessment/Évaluation:

knowledge translation (KT) tool; and (ii) will co-design, personalized and tailored care pathways or experiences (i.e., diabetes education via LearnDiabetes, peer support, virtual care, remote patient monitoring) that can be implemented for the right patient at the right time to mitigate the risk of sub-optimal outcomes.
With respect to what might be learned from the registry, it seems unlikely it will uncover new information or insights not already known. In particular, much is known from the T1D Exchange registry and other registries in the U.S. that should translate directly to youth with T1D in Canada. That registry evaluated demographic, psychosocial, socioeconomic, and clinical factors in depth in many thousands of youth with T1D. There also are other large registries in Europe and Australia that have addressed many of the same concepts.

Even if developing an algorithm to personalize care is not successful, the data integration part of the proposal may be useful by itself if easy to use and graphics are intuitive and easy to understand particularly if the app is better than existing apps such as with Glooko or Tidepool. This is the most likely potential value of this proposal.

3. **Originality of the Proposal**

The development of the TrustSphere is novel if it is successful in integrating device data with patient-reported experiences on an ongoing and sustained basis. There are other applications for device data integration (eg, Glooko, Tidepool) but the integration with patient-reported data makes this novel if it could be successfully implemented in this patient population of youth.
With respect to the objective to ‘generate knowledge regarding the heterogeneity of the lived T1D experience and subsequent outcomes that will enable and inform new precision medicine approaches for the management of T1D in children and youth’, much is already known and it seems unlikely that this proposal will uncover new information in this regard.

4. **Applicant(s)**

The NPA is very experienced with considerable expertise in type 1 diabetes. He has led an interdisciplinary team for the development and early testing of TrustSphere. He also is the NPA for a national pediatric diabetes registry and network (Canadian Pediatric Diabetes Consortium). Other lead investigators and co-applicants are also strong and form a well-rounded interdisciplinary group. One of the co-applicants is an early career researcher, one is a parent of a child with T1D, and one will serve as the project’s sex and gender champion.

5. **Environment for the Research**

**Review Type/Type d'évaluation:**	Committee Member 2/Membre de comité 2
**Name of Applicant/Nom du chercheur:**	Amed, Shazhan
**Application No./Numéro de demande:**	485828
**Agency/Agence:**	CIHR/IRSC
**Competition/Concours:**	2022-08-23 Team Grant: CIHR-JDRF Type 1 Diabetes and Precision Medicine/Subvention d'équipe : IRSC-FRDJ : Médecine de précision dans le diabète de type 1
**Committee/Comité:**	Team Grant: CIHR-JDRF Type 1 Diabetes and Precision Medicine/Subvention d'équipe : IRSC-FRDJ : Médecine de précision dans le diabète de type 1
**Title/Titre:**	Precise Treatment for Pediatric Diabetes: Providing the right care, for the right patient, at the right time, over time

Assessment/Évaluation:

The environment to conduct the research is strong.

6. Impact of the Research

The potential impact of this research is great if the researchers are successful in fully developing TrustSphere to integrate device data and patient/family reported experiences on a continual basis and in getting this technology and approach adapted widely outside of a research setting. This will be of value even if the goal to expand existing algorithms to develop personalized approaches to T1D management does not provide novel information for personalization over and above what is already known.

**Review Type/Type d'évaluation:**	Committee Member 3/Membre de comité 3
**Name of Applicant/Nom du chercheur:**	Amed, Shazhan
**Application No./Numéro de demande:**	485828
**Agency/Agence:**	CIHR/IRSC
**Competition/Concours:**	2022-08-23 Team Grant: CIHR-JDRF Type 1 Diabetes and Precision Medicine/Subvention d'équipe : IRSC-FRDJ : Médecine de précision dans le diabète de type 1
**Committee/Comité:**	Team Grant: CIHR-JDRF Type 1 Diabetes and Precision Medicine/Subvention d'équipe : IRSC-FRDJ : Médecine de précision dans le diabète de type 1
**Title/Titre:**	Precise Treatment for Pediatric Diabetes: Providing the right care, for the right patient, at the right time, over time

Assessment/Évaluation:

This application proposes to expand a diabetes data integration tool, TrustSphere, to include more data (inc for example PROMs and physical activity), expand the pilot group and develop predictive algorithms to identify undesirable outcomes and identify personalised care pathways to address these.
The group has great technological expertise, but the project seems to me to have the wrong focus. It is not too hard to identify young people who are heading for undesirable outcomes e.g. non-attendance at clinic, non-wearing of devices (as quote in the application) and high HbA1c. A complex machine learning algorithm is not required. Predictive algorithms are also not required - these individuals have trouble from the start, rather than drift into trouble. Particularly for those with the worst metabolic control, the problem is not that the don't know what to do, it is that they don't want to do it - ie disengaging with the care process. Increasing monitoring and collecting of "PROM" data may help a group with modestly raised HbA1c, but not the most at risk.

The key part of the project is phase 3B: "To Co-create *precise and personalized care pathways* to prevent undesirable outcomes anticipated via the application of predictive algorithms". The applicants suggest that an advisory board (comprising 5 youth) and a workshop series will come up with these care pathways. However, decades of effort have failed to do this, and it is not clear how they will achieve what no-one else has. The solutions are often outside of the healthcare team - e.g. changing relationships and family environments. It is also of note that they have not selected youth for the advisory board specifically from the most challenged - e.g. those with HbA1c persistently > 10%. Indeed, these individuals are unlikely to join the group until they are much older and have overcome the problem. In addition, they will deliberately not wear their technology as they don't want others to see what they are doing.

In summary this is a technologically impressive proposal but one that seems unlikely to impact those youth at the highest risk. For them, the issue is not lack of information, but lack of understanding and engagement and this is not addressed in this application in a way the that convinces that they will success where everyone else has failed.

**Review Type/Type d'évaluation:**	Committee Member 4/Membre de comité 4
**Name of Applicant/Nom du chercheur:**	Amed, Shazhan
**Application No./Numéro de demande:**	485828
**Agency/Agence:**	CIHR/IRSC
**Competition/Concours:**	2022-08-23 Team Grant: CIHR-JDRF Type 1 Diabetes and Precision Medicine/Subvention d'équipe : IRSC-FRDJ : Médecine de précision dans le diabète de type 1
**Committee/Comité:**	Team Grant: CIHR-JDRF Type 1 Diabetes and Precision Medicine/Subvention d'équipe : IRSC-FRDJ : Médecine de précision dans le diabète de type 1
**Title/Titre:**	Precise Treatment for Pediatric Diabetes: Providing the right care, for the right patient, at the right time, over time

Assessment/Évaluation:

Precise Treatment for Pediatric Diabetes: Providing the right care, for the right patient, at the right time, over time

Summary of application

Dr Amed and the project team of 16 others plans to create a single site called TrustSphere to house data of all types for patients with T1D and they are currently testing version 1 of the platform with 50 patients. This site, which will be accessible for patients, parents, caregivers and HCPs has several purposes: to gather all the relevant data in one place and include information from questionnaires, to allow for real time interventions and corrections to be made to improve outcomes for the patient, and to provide data from consenting patients for a registry and for research studies. The stated goal is to predict which patients are more at risk of undesirable outcomes, and to create personalized care pathways.

There are 3 phases of the proposal:

-include data from more devices (a range of glucometers, sensors, pumps, activity and sleep trackers) as well as patient-reported questionnaires related to quality of life and diabetes distress

-expand the real-world study to 150 users of TrustSphere and create a link with a patient registry

-analyze the data with machine learning tools to predict undesirable outcomes and create care pathways for individual patients.

Strengths

The strengths of the application are:

-an excellent and qualified group of investigators having diverse areas of relevant expertise. They also have strong experience in publishing, giving presentations and knowledge translation

-existence of a prototype of the TrustSphere platform which is being tested currently with 50 users

-prior information on how good machine learning will be for predicting poor outcomes (from a US database) with 93% specificity, but only 50% PPV for a modest rise in HbA1c.

-inclusion of a person with lived experience of diabetes (PWLE parent Ms Miller) on the Advisory board of the study

**Review Type/Type d'évaluation:**	Committee Member 4/Membre de comité 4
**Name of Applicant/Nom du chercheur:**	Amed, Shazhan
**Application No./Numéro de demande:**	485828
**Agency/Agence:**	CIHR/IRSC
**Competition/Concours:**	2022-08-23 Team Grant: CIHR-JDRF Type 1 Diabetes and Precision Medicine/Subvention d'équipe : IRSC-FRDJ : Médecine de précision dans le diabète de type 1
**Committee/Comité:**	Team Grant: CIHR-JDRF Type 1 Diabetes and Precision Medicine/Subvention d'équipe : IRSC-FRDJ : Médecine de précision dans le diabète de type 1
**Title/Titre:**	Precise Treatment for Pediatric Diabetes: Providing the right care, for the right patient, at the right time, over time

Assessment/Évaluation:

-links with industry and government bodies which should facilitate inclusion of all the data sources and help with ensuring requirements for privacy are upheld.

Weaknesses

The weaknesses which I have identified are in the form of questions which are not clearly addressed in the proposal:

-Feasibility: How will physicians and other HCPs treating these patients be able to offer real-time advice and modifications to the treatment paradigm? Is there a way for their time to be reimbursed by the provincial healthcare system? Will they receive alerts when one of their patients is "off track"? How will the team get buy-in from endos not currently involved in the project (for example an endo in the interior of BC who treats mostly people with T2D and is over-burdened already)?

-Practical issues: how to combat data and questionnaire fatigue among patients and parents? How to alert the platform about device failure/error within the system, to avoid false data being collected? What is the minimum time that a user must be involved to collect valid data?

-Applicability: How to involve low-income families? Can they be sponsored? How will First nations, Inuit and Metis patients be recruited?

Durability: How long does the effect of using TrustSphere last if someone stops or takes a break from using it?

Is it generalizable? Can it be used for patients with T2D? They may benefit the most due to poorer compliance. Can it work as well in other populations outside of the greater Vancouver area?

Does it meet Precision Medicine goals: How will environmental factors be captured? It seems that the population to be studied will be quite homogenous. Without genetic and molecular factors, how will the data be analyzed to address the ability for the site to predict the effect of heterogeneity in these variables and create care pathways for a greater portion of the people with T1D in Canada?

Budget

**Review Type/Type d'évaluation:**	Committee Member 4/Membre de comité 4
**Name of Applicant/Nom du chercheur:**	Amed, Shazhan
**Application No./Numéro de demande:**	485828
**Agency/Agence:**	CIHR/IRSC
**Competition/Concours:**	2022-08-23 Team Grant: CIHR-JDRF Type 1 Diabetes and Precision Medicine/Subvention d'équipe : IRSC-FRDJ : Médecine de précision dans le diabète de type 1
**Committee/Comité:**	Team Grant: CIHR-JDRF Type 1 Diabetes and Precision Medicine/Subvention d'équipe : IRSC-FRDJ : Médecine de précision dans le diabète de type 1
**Title/Titre:**	Precise Treatment for Pediatric Diabetes: Providing the right care, for the right patient, at the right time, over time

Assessment/Évaluation:

The budget has a limited fund for inclusion of PWLE in the advisory board meetings, and there is a small honorarium for the people who respond to the planned interviews in phase 1 and the study participants in Phase 2. This is a small fraction of the overall budget (<2%). There should be a provision for more consultation with PWLE in years 3 and 4 when the findings are going to be disseminated and care pathways are being developed. It will be important to verify with PWLE whether they understand the outcomes and the advice that will be provided. Additional consultation would ensure that the study answers questions that are meaningful to future users.

The project is well thought out and the plan is rigorous, and it addresses an important gap in care for children with T1D. However, it does not go far enough to meet the stated goal of the precision medicine mandate. It also does not go far enough in providing something original which is not currently being captured, albeit in multiple other platforms.

Review Type/Type d'évaluation: Committee Member 5/Membre de comité 5
Name of Applicant/Nom du chercheur: Amed, Shazhan
Application No./Numéro de demande: 485828
Agency/Agence: CIHR/IRSC
Competition/Concours: 2022-08-23 Team Grant: CIHR-JDRF Type 1 Diabetes and Precision Medicine/Subvention d'équipe : IRSC-FRDJ : Médecine de précision dans le diabète de type 1
Committee/Comité: Team Grant: CIHR-JDRF Type 1 Diabetes and Precision Medicine/Subvention d'équipe : IRSC-FRDJ : Médecine de précision dans le diabète de type 1
Title/Titre: Precise Treatment for Pediatric Diabetes: Providing the right care, for the right patient, at the right time, over time

Assessment/Évaluation:

1. Research Question

- Overall, the *idea* presented here is excellent and it represents a smart solution to a real challenge. Data have the potential to optimize care for each patient, but those data are currently disparate, underutilized, and could easily be leveraged to improve health outcomes. This approach closely mirrors what we see in other sectors (e.g., consumer goods) insofar as tailoring recommendations to each user, rather than across entire populations.

2. Research Approach

- There are lots of reasons to think this project idea has potential, particularly in the promise of data to predict outcomes more accurately and dynamically than a person can.
- There are some pragmatic concerns; the biggest is with respect to user engagement, on which the success of this idea may ultimately hinge. How do we know health care workers or patients/families will engage with the application? Will they understand what their incentive to do so is, or is there any reason they *need* to engage? There are lots of examples of apps that solve real problems, but users simply don’t engage with them. There is also potential for challenges when users engage with algorithmic recommendations. Will users trust algorithmic outputs and recommendations with respect to diabetes care? Algorithmic trust is a key issue in app development currently (see research of Jennifer Logg at GWU, for example). Some of the qualitative interviews (Ph 3B, Step 2) may be useful here.
- On another note, is there a way to ensure data quality, particularly as it’s collected from families? Some of these data sound like they may be qualitative and/or self-reported (e.g., activity levels). High quality data need to be ensured prior to use in any machine learning process (the ‘garbage in – garbage out’ principle).
- Is 150 patients/families in Ph2enough data to support the key questions being asked here? The researchers address that this is of concern but don’t provide a specific alternative or other recommendation.

3. Originality of the Proposal

- I will defer to other committee members who are more familiar with the literature

4. Applicant(s)

- No major notes regarding applications themselves

5. Environment for the Research

- No major flags regarding the environment for this research

6. Impact of the Research

- The potential for impact with this project is substantial. If adopted and scaled well, it could change standard courses of treatment for PLWT1D in Canada.
- What is the capacity of this project to be scaled elsewhere? Given the funding levels, it would be interesting to at

**Review Type/Type d'évaluation:**	Committee Member 5/Membre de comité 5
**Name of Applicant/Nom du chercheur:**	Amed, Shazhan
**Application No./Numéro de demande:**	485828
**Agency/Agence:**	CIHR/IRSC
**Competition/Concours:**	2022-08-23 Team Grant: CIHR-JDRF Type 1 Diabetes and Precision Medicine/Subvention d'équipe : IRSC-FRDJ : Médecine de précision dans le diabète de type 1
**Committee/Comité:**	Team Grant: CIHR-JDRF Type 1 Diabetes and Precision Medicine/Subvention d'équipe : IRSC-FRDJ : Médecine de précision dans le diabète de type 1
**Title/Titre:**	Precise Treatment for Pediatric Diabetes: Providing the right care, for the right patient, at the right time, over time

Assessment/Évaluation:

least consider what that might look like as a learning from the initial research. Scaling itself would likely be out of the question for this proposal but identifying things that worked well or didn’t during the process would be helpful.
