# Supplementary material for: Real-World Implementation and Evaluation of a Digital Health Platform for Children Living With Type 1 Diabetes: Protocol for a Real-World Implementation Study
Source: JMIR Res Protoc. 2026 Oct 30;15:e107634. doi: 10.2196/107634 (PMC13722103; doi:10.2196/107634)
Supplement: Supplementary file 2 [file resprot-v15-e107634-s001.docx]

**Supplementary Table 1**

| **Patient-Level Data** | | |
| --- | --- | --- |
| **BC-PDR Variable** | **Type and Units** | **BC-PDR Description** |
| **BC-PDR Patient ID** | Integer | Historical Unique Patient Identifier: Patient Identifier   When consent was required, this ID was assigned to patients who had consented to participate in the BC-PDR (not included in CDRH) |
| **PRISM Patient ID** | Text | This Unique Patient ID will be generated in PRISM. Will be used to deidentify or anonymize for research purposes, including SWEET, CAPACITY, and PHN Linkage |
| **Email** | Text | Email is collected for PROMs and PREMs (not included in CDRH) |
| **Sex** | Integer | Biological Sex of Patient  1,Female \| 2,Male \| 3, Unknown |
| **Patient's date of birth** | Text (YYYY-MM-DD) | Patient's date of birth (truncated to birth month and year for CDRH) |
| **Date of Diabetes Diagnosis** | Text (YYYY-MM-DD) | Date of Diabetes Diagnosis |
| **Ethnicity** | Integer | Ethnicity as defined by the patient or patient's caregiver. More than one can be selected  1, Arab \| 2, Black \| 3, Chinese \| 4, Filipino \| 5, Japanese \| 6, Korean \| 7, Latin American \| 8, South Asian\| 9, Southeast Asian \| 10, West Asian \| 11, White \|12, First Nations \| 13, Inuit \| 14, Metis \| 15, Unknown \| 16, Other (specified) \| 17, Prefer not to disclose  *Note ethnicity will be collected as part of PROMs/PREMs |
| **Ethnicity (Other)** | Text | If Ethnicity is "Other", specify |
| **Type of Diabetes** | Integer | 1, Type 1 Diabetes \| 2, Type 2 Diabetes \| 3, Cystic Fibrosis Related Diabetes \| 4, Medication Related Diabetes \| 5, MODY \| 6, Neonatal Diabetes \| 7, Other Diabetes \| 999, Missing |
| **Type of Diabetes (Other, specify)** | Text | If Type of Diabetes is "Other", specify |
| **Patient Deceased** | Integer | 1, Yes \| 0, No |
| **Cause of Death** | Integer | Cause of Death  1, DKA \| 2, Hypoglycemia \| 3, Drug Overdose \| 4, Suicide \| 5, Accident \| 6, Underlying non-diabetes condition \| 7, Other |
| **Cause of Death (Other)** | Text | If other is selected, please include cause of death |
| **Chronic Comorbidities** | Integer | Additional condition/disease that the patient has been diagnosed with. Listed either by disease name or ICD-10 code  1, Addison disease (E27.1) \| 2, celiac disease (K90.0) \| 3, hyperthyroidism (E05.0)\|4, hypothyroidism (E06.3) \| 5, Down syndrome (Q90) \| 6, Turner syndrome (Q96) \| 7, PCOS (E28.2) \| 8, TPO+ (R76.0) \| 9, microalbuminuria (N08.3) \| 10, retinopathy (H36.0) \| 11, Prader–Willi syndrome (Q87.1) \| 12, hypopituitarism (E23.0) \| 13, rheumatoid arthritis (M08.0) \| 14, inflammatory bowel disease (K50) \| 15, nephropathy (N04.9) \| 16, tTG+ (R76.0) \| 17, autism (F84.0) \| 18, gender dysphoria (F64.2) \| 19, malignancy: leukemia/lymphoma (C96.9) \| 20, malignancy: solid tumour (C80) \| 21, s/p kidney transplant (Z94.0) \| 22, MASLD (K76.0) \| 23, OSA (K47.3) \| 24, ADHD (F90) \| 25, Anxiety (F41.1) \| 26, Depression (F32.X) \| 27, Learning Disability (F81.9) \| 28, Dyslipiedmia (E78.5) \| 29, Hypertension (I10) \| 30, NAFLD (K76.0) \| 31, Diabetic neuropathy (G63.2) \| 32, Gastroparesis (K31.84) \| 33, Foot Ulcer- Type 1 (E10.621) \| 33, Foot Ulcer- Type 2 (E11.621) |
| **Presentation at Onset** | Integer | Select the most severe presentation applicable  Presentation of Diabetes at Onset  1, DKA \| 2, Hyperglycemia \| 3, By Screening \| 4, HHS \| 5, Other \| 999, Missing |
| **Presentation at Onset- Coma Specification** | Integer | 1, With Coma \| 2, Without Coma |
| **HbA1c on Diagnosis** | Decimal (%)  (1-2 decimal) | HbA1c measurement recorded during the first 10 days after diagnosis  Minimum value: 3.0, Maximum value: 20 |
| **GAD65 Date** | Text (YYYY-MM-DD) | Date of GAD65 |
| **GAD65** | Integer | 0, Negative \| 1, Positive \| 888, Not Available   From the EMR, results can be directly pulled as positive/negative or as discrete values which can be harmonized to positive/negative.  Negative defined as  Positive defined as |
| **Antibodies Testing Date** | Text (YYYY-MM-DD) | Date of Antibody Testing |
| **ZnT8** | Integer | 0, Negative \| 1, Positive \| 888, Not Available   From the EMR, results can be directly pulled as positive/negative or as discrete values which can be harmonized to positive/negative.  Negative defined as  Positive defined as |
| **IA-2** | Integer | 0, Negative \| 1, Positive \| 888, Not Available   From the EMR, results can be directly pulled as positive/negative or as discrete values which can be harmonized to positive/negative.  Negative defined as  Positive defined as |
| **Insulin Antibody** | Integer | 0, Negative \| 1, Positive   From the EMR, results can be directly pulled as positive/negative or as discrete values which can be harmonized to positive/negative.  Negative defined as  Positive defined as |
| **C peptides** | Integer (pmol/l) | Less than 15, undetectable  888, Not Available |

| **Visit-Level Data** |  |  |
| --- | --- | --- |
| **BC-PDR Variable** | **Type and Units** | **BC-PDR Description** |
| **BC-PDR Patient ID** | Text | Historical Unique Patient Identifier: Patient Identifier   When consent was required, this ID was assigned to patients who had consented to participate in the BC-PDR (not included in CDRH) |
| **PRISM Patient ID** | Text | This Unique Patient ID will be generated in PRISM. The ID will be used to deidentify or anonymize for research purposes, including SWEET, CAPACITY, PHN Linkage, and any data access requests. |
| **Date of Visit** | Text (YYYY-MM-DD) | Patient's date of visit |
| **Type of Visit** | Integer | 1, Clinic \| 2, Lab \| 3, Virtual  What type of visit does the record represent. This field can be derived from existing variables in the EMR and will be generated during harmonization |
| **Clinic** | Integer | 1, BC Children's Hospital \| 2, Kamloops DEC \| 3, Kelowna DEC \| 4, Vernon DEC \| 5, Penticton DEC \| 6, Trail DEC \| 7, Nelson DEC \| 8, Cranbrook DEC \| 9, Creston DEC \| 10, Abbotsford Regional Hospital and Cancer Centre \| 11, Surrey Memorial Hospital \| 12, Campbell River DEC \| 13, Nanaimo DEC \| 14, Victoria DEC \| 15, Prince George Diabetes Clinic \| 16, North Shore Chronic Disease Clnic \| 17 Other |
| **Clinic (Other)** | Text | If Clinic="other", please specify |
| **Postal Code** | Text | Postal code is recorded at diagnosis and at each visit. This will be automatically collected via PRISM (not included in CDRH) |
| **Height** | Decimal (cm)  1 decimal place | Height of patient at day of visit |
| **Weight** | Decimal (kg)  1 decimal place | Weight of patient at day of visit |
| **BMI** | Decimal (Kg/m2)  1 decimal place | BMI of patient at day of visit |
| **Blood pressure- systolic** | Integer (mmHG) | Systolic blood pressure at day of visit |
| **Blood pressure- diastolic** | Integer (mmHG) | Disatolic blood pressure at day of visit |
| **POC HbA1c** | Decimal (%)  1 decimal place | HbA1c measurements recorded at day of visit. |
| **Lab HbA1c** | Decimal (%)  1 decimal place | HbA1c measurements recorded within the last 45 days of a clinic visit. |
| **Cholesterol** | Decimal (mmol/L)  1- 2 decimal | Fasting or non-fasting cholesterol recorded at day of clinic visit or lab visit. |
| **HDL cholesterol** | Decimal (mmol/L)  1- 2 decimal | HDL cholesterol recorded at day of clinic visit or lab visit |
| **LDL cholesterol** | Decimal (mmol/L)  1- 2 decimal | LDL cholesterol recorded at day of clinic visit or lab visit. |
| **Triglyceride** | Decimal (mmol/L)  1- 2 decimal | Triglycerides recorded at day of clinic visit or lab visit. |
| **TSH** | Decimal (mU/L)  1 or 2 decimal | Thyroid stimulating hormone levels recorded at day of clinic visit or lab visit. |
| **Free T4** | Decimal (pmol/L)  1- 2 decimal | Free thyroxine levels recorded at day of clinic visit or lab visit. |
| **Thyroid Peroxidase Antibody** | Decimal (U/mL)  1 decimal | Thyroid Peroxidase Antibody recorded at day of clinic visit or lab visit. |
| **Celiac Antibodies (ttG)** | Decimal (U/mL)  1 decimal | Celiac Antibodies recorded at day of clinic visit or lab visit. |
| **Nephropathy (ACR Lab Test)** | Decimal (mg/mmol)  1- 2 decimal | Albumin to creatinine Ratio Lab Test recorded at clinic visit or lab visit. |
| **eGFR** | Decimal (mL/min/1.73 m2)  1 decimal | Estimated Glomerular Filtration Rate recorded at day of clinic visit or lab visit |
| **Is patient on pump** | integer | At the time of the visit, whether the patient is on pump  0, no \| 1, yes |
| **Number of injection time points per day** | Integer | The patient's reported number of injection time points per day at time of visit  1, Once daily \| 2, BID (2 times) \| 3, TID (3 times) \| 4, MDI (4 times or more) \| 5, Once Weekly \| 6, Zero \| 7, Other  Will only be available if "patient on pump"=no |
| **Number of injection time points per day (specify)** | text | If "Number of Injections time points per day"=other, please specify |
| **Type of pump therapy** | Integer | Type of pump therapy at time of visit  1, Non Hybrid Closed Loop \| 2, Hybrid Close Loop \| 3, DIY ~~\|~~ 4, Other |
| **Type of pump therapy, Other (specify)** | text | If "type of pump therapy=4", please specify |
| **Total Daily Insulin dose** | Units per day (1 decimal place) | The patient's daily insulin dose at time of visit.  Will only be available if "patient on pump"=yes. |
| **Daily Basal Insulin dose** | Units per day (1 decimal place) | The patient's daily insulin dose at time of visit.  Will only be available if "patient on pump"=yes. |
| **Daily Prandial Insulin dose** | Units per day (1 decimal place) | The patient's daily insulin dose at time of visit.  Will only be available if "patient on pump"=yes. |
| **Basal Percent** | Integer (%) | The percentage of total daily insulin that is basal insulin  Will only be available if "patient on pump"=yes. |
| **Bolus Percent** | Integer (%) | The percentage of total daily insulin that is bolus insulin  Will only be available if "patient on pump"=yes. |
| **Bolusing routine** | Integer | Timing of pre-meal insulin is delivered  1,Before Meals \| 2, After Meals \| 3, Before and After Meals \| 4, Other  Available if "patient on pump"=yes OR no |
| **Bolusing Routine (Other)** | Text | If Bolusing routine=4, please specify |
| **Average boluses per day** | Decimal (1 decimal place) | Will only be available if "patient on pump"=yes. |
| **Sensor Use** | Integer | If patient is using a sensor, the patient reports that they use the sensor on a regular basis since the last visit that was entered into the registry   0, no \| 1, yes on a regular basis (Defined as more than 80% of the days since the last visit). |
| **Number of Self-Monitoring of Blood Glucose (SMBG) per day** | Integer | The patient's reported average daily number of SMBG at time of visit. If given a range, the larger number will be used (e.g. 4-8 checks per day, enter 8).   Will only be filled if "sensor use"= no |
| **Sensor Usage (%)** | Integer (%) | % of Days with CGM Readings (Max=100%)  Will only be filled if "sensor use"= yes |
| **Time in range** | Integer (%) | % Time spent in the target range between 3.89 and 9.99 mmol/L during the last 2 weeks (Max=100%)  Will only be filled if "sensor use"= yes |
| **Time above range** | Integer (%) | % Time spent above 9.99 mmol/L during the last 2 weeks (Max=100%)  Will only be filled if "sensor use"= yes |
| **Time below range** | Integer (%) | % Time spent below 3.89 mmol/Lduring the last 2 weeks (Max=100%)  Will only be filled if "sensor use"= yes |
| **GMI** | Decimal  1 decimal place | A1C from Sensor  Will only be filled if "sensor use"= yes |
| **Coefficient of variation** | Integer | A standardized measure that assesses the magnitude of glucose variability  Will only be filled if "sensor use"= yes |
| **Patient Reviewed CGM data** | Integer | Does patient review CGM data between visit?  1, Yes \| 0, No  Will only be filled if "sensor use"=yes |
| **Non-insulin Diabetes Medication** | Integer | 1, Metformin \| 2, GLP-1 Agonist \| 3, SGLT-2 Inhibitors \| 4, Sulfonylurea \| 5, Thiazolidinedione \| 6, Other |
| **Non-insulin Diabetes Medication (Other)** | Text | If "Non-insulin Diabetes Medication"=Other, specify |
| **Severe Hypoglycemia** | Integer (Number of events) | The number of severe hypoglycemia events since the last visit that was entered into the registry. Event in this case is defined as impaired consciousness or seizure. |
| **DKA leading to hospitalisation** | Integer (Number of events) | The number of events since the last visit that was entered into the registry (DKA, excluding DKA at diabetes onset). |
| **Screening of Retinopathy** | Integer | If a retinopathy screening, an eye exam within the past year, occurred since the last visit that was entered into the registry.   Screening for retinopathy  1, Yes \| 0, No   When calculating screening rates for retinopathy only include patients with T1D who are 15 years and older and have been living with diabetes for at least 5 years. |
| **Insulin Adjusted since last visit** | Integer | 1, Yes, by patient and caregiver \| 0, No |
| **Missed school** | Integer (number of days) | Amount of days patient missed school due to diabetes complications since their last visit |
| **Screen time** | Decimal (Hours per day)  1 decimal place | A daily average of the amount of time the patient spent on a screen since their last visit |
| **Physical activity** | Decimal (Hours per day)  1 decimal place | A daily average of the amount of time the patient spent being physically acitive their last visit |
| **Sleep habits** | Decimal (Hours per day)  1 decimal place | A daily average of the amount of time the patient sleeps since their last visit |
